# Fenofibrate restores glutamatergic and dopaminergic homeostasis in the nucleus accumbens and reduces alcohol relapse in rats

**DOI:** 10.3389/fphar.2026.1792426

**Published:** 2026-03-23

**Authors:** Eduardo Isla, Ignacio Gutiérrez-Vega, María Elena Quilaqueo, Mario Herrera-Marschitz, María Elena Quintanilla, Paola Morales, Diliana Pérez-Reytor, Mario Rivera-Meza, Eduardo Karahanian

**Affiliations:** 1 Institute of Biomedical Sciences, Faculty of Health Sciences, Universidad Autónoma de Chile, Santiago, Chile; 2 Research Center for the Development of Novel Therapeutic Alternatives for Alcohol Use Disorders, Santiago, Chile; 3 Department of Pharmacological and Toxicological Chemistry, Faculty of Chemical Sciences and Pharmacy, Universidad de Chile, Santiago, Chile; 4 Interdisciplinary Nucleus of Pharmacology & Immunology, Institute of Biomedical Sciences ICBM, Faculty of Medicine, Universidad de Chile, Santiago, Chile

**Keywords:** alcohol use disorder, alcoholism, fibrates, PPAR alpha, UChB rats

## Abstract

Alcohol use disorder (AUD) is associated with dysregulation of glutamatergic and dopaminergic signaling within the nucleus accumbens (NAc), contributing to withdrawal syndrome, craving, and relapse. Fenofibrate, an agonist of the peroxisome proliferator-activated receptor alpha (PPARα), reduces alcohol intake; however, its effects on NAc neurotransmission and the relative contributions of central versus peripheral mechanisms remain unclear. Here, we investigated whether fenofibrate administration initiated during alcohol withdrawal and continued throughout reaccess reduces relapse drinking, and whether this effect involves normalization of glutamate and dopamine in the NAc. Fenofibrate treatment during withdrawal produced a sustained reduction in alcohol consumption throughout a 14-day relapse period in high-drinker female rats. This effect was abolished by systemic co-administration of the PPARα antagonist GW6471. Fenofibrate increased hepatic catalase activity and upregulated the glutamate transporter GLT-1 in the NAc, while dopamine transporter (DAT) protein levels remained unchanged. When GW6471 was administered intracerebroventricularly to block central PPARα activation selectively, the reduction in alcohol intake was only partially attenuated, indicating that approximately one-third of fenofibrate’s effect is centrally mediated, whereas two-thirds result from peripheral mechanisms. No-net-flux microdialysis showed that fenofibrate enhanced uptake rates for both glutamate and dopamine in the NAc. Steady-state extracellular glutamate levels were unchanged, whereas extracellular dopamine levels were significantly reduced, consistent with increased functional DAT activity. These findings demonstrate that fenofibrate reduces relapse-like alcohol consumption through a combination of peripheral and CNS PPARα-dependent mechanisms, restoring key aspects of glutamatergic and dopaminergic homeostasis in the NAc, highlighting PPARα activation as a promising therapeutic strategy for AUD.

## 1 Introduction

Alcohol use disorder (AUD) is a major global public health issue, associated with high morbidity and significant social and economic consequences (World Health Organization, 2024). This condition is characterized by the development of tolerance to alcohol’s effects, increased consumption, loss of control over drinking, and the emergence of physical dependence (Grant et al., 2015). Current treatments for AUD include cognitive/behavioral interventions, pharmacological approaches, and/or a combination of both. FDA-approved medications such as disulfiram, naltrexone, and acamprosate have shown limited efficacy, particularly in preventing medium- and long-term relapses (Agabio et al., 2024; Maisel et al., 2013; Plosker, 2015). This underscores the need to identify more effective treatment strategies.

Withdrawal and relapse in AUD are closely linked to dysregulation of the brain’s reward system, primarily mediated by the neurotransmitters dopamine and glutamate. Chronic alcohol consumption disrupts the homeostasis of these systems, promoting drug-seeking behavior and compulsive alcohol use. On one hand, alcohol increases extracellular glutamate levels in several brain regions, including the nucleus accumbens (NAc), through various mechanisms, most notably by downregulating the astrocytic glutamate transporter GLT-1, leading to excitotoxicity and neuronal dysfunction (Holmes et al., 2013; Villavicencio-Tejo et al., 2021). On the other hand, chronic alcohol consumption induces neuroadaptations of dopaminergic transmission in the brain. Long-term exposure to alcohol leads to a series of neurobiological adaptations that impair dopaminergic regulatory mechanisms of reward and reinforcement (Ma and Zhu, 2014). Additionally, but no less critical, neurochemical alterations in AUD include GABAergic, serotonergic, and opioid systems (Prajapati et al., 2020). Studies suggest alterations in cortical GABA levels that may reflect compensatory or maladaptive responses to sustained alcohol exposure and withdrawal; for example, it was reported decreased cortical GABA levels in regions such as the anterior cingulate cortex (ACC) and prefrontal areas, consistent with a dysregulation of GABAergic tone in AUD compared to controls (Spitta et al., 2024). About the serotonergic system, chronic AUD is associated with decreased central serotonergic transmission in cortical/striatal areas in several human and animal studies, consistent with reduced serotonergic tone during prolonged exposure and increased relapse risk (Marcinkiewcz et al., 2016). Finally, chronic alcohol exposure leads to persistent adaptations in the endogenous opioid system. Many studies report upregulation or altered turnover of opioid peptides and changes in receptor signaling in reward-related circuits, intimately linked to dopamine and GABA transmission in mesolimbic pathways; such adaptations correlate with sustained drinking and relapse vulnerability (Prajapati et al., 2020).

Research by Volkow et al. (2011) demonstrated that drugs of abuse initially elevate dopamine levels in the nucleus accumbens. Still, these dopamine surges—and the associated feelings of reward—become dampened in the person with a substance use disorder. Crucially, when drug-related cues are presented, addicted subjects exhibit robust dopamine release. The authors suggest that these conditioned responses—reflecting the anticipation of the drug’s effects—combine with the drug’s pharmacological actions to pursue the expected reward, thereby promoting relapse and sustaining drug-taking behavior. Volkow’s hypothesis suggests that pharmacological modulation of glutamatergic and dopaminergic signaling represents a promising therapeutic strategy for the treatment of AUD.

In this context, fenofibrate, an agonist of the peroxisome proliferator-activated receptor alpha (PPARα), has been proposed as a potential treatment for AUD. Fenofibrate reduces voluntary alcohol consumption in animal models by both peripheral and central mechanisms. Peripherally, fenofibrate increases hepatic catalase activity, thereby accelerating accumulation of acetaldehyde in the bloodstream, which induces dysphoric effects that contribute to reduced alcohol intake (Karahanian et al., 2014; Muñoz et al., 2020; Rivera-Meza et al., 2017). At the central level, fenofibrate exerts neuroprotective effects by modulating neuroinflammation and possibly glutamate homeostasis. Specifically, fenofibrate can inhibit NF-κB activation, reduce proinflammatory cytokine production, and normalize GLT-1 expression, potentially reducing extracellular glutamate levels and attenuating glutamatergic hyperactivity in the NAc (Ibáñez et al., 2023; Villavicencio-Tejo et al., 2021). Although direct research linking fenofibrate to dopamine modulation in the context of AUD is limited, emerging evidence suggests a plausible connection: fenofibrate has been shown not only to reduce alcohol consumption but also saccharin intake in animal models (Rivera-Meza et al., 2017), suggesting a broader influence on reward-related behaviors. This effect on the brain’s reward circuitry could stem from fenofibrate’s impact on glutamate homeostasis. Glutamate, the primary excitatory neurotransmitter in the brain, plays a crucial role in regulating dopamine release and signaling within the mesolimbic reward pathway, a key circuit implicated in addiction (Ma and Zhu, 2014). Given the close interconnection between these neurotransmitter systems, it is conceivable that the effects of fenofibrate on glutamate involve dopaminergic signaling, thereby increasing dopamine release. Another possibility is that fenofibrate alters the activity of the dopamine transporter (DAT), which reuptakes dopamine from the synaptic space. Fenofibrate’s neuroprotective properties are supported by its potential to modulate dopaminergic pathways (Barbiero et al., 2022; De Felice et al., 2019). Fenofibrate has also been associated with improvements in depression-related behaviors by enhancing dopamine activity in the mesolimbic pathway, a critical region for reward and motivation (León et al., 2025; Scheggi et al., 2016), suggesting a potential role in restoring dopaminergic balance. However, no direct evidence supports these suggestions, leaving it to be demonstrated that fenofibrate can indeed restore the glutamatergic and/or the dopaminergic imbalance induced by chronic alcohol consumption.

Although PPARα activation has been associated with alterations in reward-related behaviors and dopaminergic dysfunction in other pathological conditions (Scheggi et al., 2022), the mechanisms through which fenofibrate could modulate dopamine signaling in AUD remain largely hypothetical. Indirect evidence has suggested possible effects on DAT trafficking, transporter kinetics, or presynaptic dopamine release, but these mechanisms have not been experimentally demonstrated in models of chronic alcohol exposure. In this context, the present study was designed to clearly distinguish between direct experimental observations -such as the effects of fenofibrate on glutamatergic and dopaminergic tone in the nucleus accumbens assessed through no-net-flux microdialysis- and mechanistic interpretations that remain inferential. We wanted to test the hypothesis that fenofibrate treatment during withdrawal would enhance glutamate and dopamine clearance in the NAc via increased GLT-1 and DAT expression and function, respectively, and that these effects would be mediated through PPARα-dependent mechanisms involving both peripheral and central components. We evaluated whether this treatment normalizes neurotransmitter homeostasis in ways that may explain its central effects on reducing alcohol intake by administering fenofibrate during alcohol withdrawal in rats with a history of chronic drinking. Moreover, because fenofibrate acts both peripherally and centrally, we sought to determine the relative contributions of each compartment to its behavioral effects by selectively blocking PPARα activation in the CNS via intracerebroventricular injection of a specific antagonist.

## 2 Materials and methods

### 2.1 Animals and treatments

High-drinker UChB (University of Chile Bibulous) rats derived from the Wistar strain and selectively bred for high alcohol intake (Israel et al., 2017) were used. Two-month-old female rats were housed in individual cages in temperature-controlled rooms under a regular 12-h light/12-h dark cycle. Rats were offered a free choice between two bottles: one containing 10% (v/v) alcohol (Ethanol, Merck) solution and the other containing water for 45 days. Food (Mardones rat formula, Alimentos Cisternas, Santiago, Chile) and water were provided *ad libitum*, and the volumes of water and alcohol were recorded daily. After 45 days of alcohol consumption, the weight of the animals averaged 242.1 ± 18.9 g. The decision to use female rats in this study is based on compelling evidence from UChB and other rat lines selectively bred for elevated alcohol consumption, such as the high-alcohol-drinking-2 (HAD-2) and Sardinian alcohol-preferring (sP) lines, where females consistently demonstrated higher and stable alcohol consumption than males, as well as reduced inter-individual variability (Dhaher et al., 2012; Israel et al., 2017; Loi et al., 2014).

In Experiment 1, rats were given continuous free-choice access to alcohol and water for 45 days. After this period, animals were deprived of alcohol for 14 days while food and water remained available *ad libitum*. They were then randomly assigned to four groups (n = 6 per group). During the last 5 days of alcohol withdrawal, each group received one of the following daily treatments:

- Group I–Vehicle control: intraperitoneal (i.p.) dimethyl sulfoxide (DMSO; 0.1 mL/100 g body weight) (Ibáñez et al., 2023) plus water by gavage (1 mL/150 g body weight) (Rivera-Meza et al., 2017), corresponding to the vehicles for GW6471 and fenofibrate, respectively.
- Group II–Fenofibrate: micronized fenofibrate (Fibronil®, Royal Pharma, Santiago, Chile) at 50 mg/kg/day by gavage (re-suspended in water) (Rivera-Meza et al., 2017), plus DMSO i.p. vehicle.
- Group III–Fenofibrate + GW6471: fenofibrate 50 mg/kg/day by gavage plus GW6471 (1 mg/kg/day, i.p.; PPARα antagonist, MyBioSource, San Diego, CA, USA) dissolved in DMSO (Ibáñez et al., 2023).
- Group IV–GW6471: GW6471 1 mg/kg/day i.p. dissolved in DMSO, plus water vehicle by gavage.

On day 60, after finishing the 14 days of abstinence (which included the 5 days of drug treatment), re-access to alcohol was allowed for a period of 14 days to assess relapse-like alcohol consumption. During the 14 days of alcohol re-access, the treatments initiated at the abstinence period were kept on, up to the time animals were anesthetized with 5% isofluorane and killed by decapitation to obtain brain and liver samples, for the determination of GLT-1 and DAT expression levels in NAc and catalase activity in the liver, respectively.

In Experiment 2, rats underwent the same alcohol consumption protocol and subsequent withdrawal period described for Experiment 1. In this experiment, however, GW6471 was administered intracerebroventricularly (i.c.v.) instead of intraperitoneally, to block fenofibrate’s actions specifically in the CNS and thereby distinguish its central from peripheral effects on alcohol intake. During the last 5 days of withdrawal, animals received daily treatments of fenofibrate (50 mg/kg/day by gavage) and/or GW6471 (1 µg in 1 µL DMSO, i.c.v.). To enable i.c.v. administration, an intracerebroventricular cannula (cat. CXG-4, AgnThos, Sweden) was implanted into the left lateral ventricle on the first day of abstinence (coordinates: AP −0.8, ML +1.6, DV −3.6 from bregma) (Paxinos, C, Watson, 2007). UChB rat line is equivalent to stereotaxic measures than the Wistar rat line, on which the Atlas by Paxinos and Watson is based. The cannula was fixed to the skull with dental acrylic secured by three 1 mm screws (cat. MCS1x2, AgnThos, Sweden). Control animals received the corresponding vehicles (1 µL DMSO i.c.v. and water by gavage). On the day after the treatment period ended, alcohol was reintroduced for 24 h, and intake was recorded.

The animal study protocol was approved by the Bioethics Committee on Animal Research, Faculty of Medicine, Universidad de Chile (Protocol 22543-MED-UCH).

### 2.2 Microdialysis, dopamine, and glutamate chromatographic detection

In experiment 3, the rats were subjected to the same alcohol consumption regimen, followed by withdrawal and fenofibrate and/or GW6471 treatments as in experiment 1. In this experiment, a naïve group was included that received no treatment. Three days before the end of the withdrawal phase, rats were anesthetized with a mixture of air and isoflurane delivered via a nose-fitted mask and positioned in a Kopf stereotaxic apparatus with the skull aligned. A guide cannula (cat. AT9.14.iC, AgnThos, Sweden) was stereotaxically implanted into the left NAc (AP +1.7 mm, ML +0.7 mm, DV −8.0 mm) (Paxinos andWatson, 2007) and secured firmly to the skull. On the day following the end of abstinence, a microdialysis probe (cat. AT9.14.2, AgnThos, Sweden) was inserted into the NAc through the guide cannula and connected to a microdialysis system (34 × 40 mm; CMA/Microdialysis AB, Stockholm, Sweden) equipped with a perfusion setup. In awake animals, probes were perfused with artificial cerebrospinal fluid (aCSF) followed by increasing concentrations of glutamate (0 μM, 0.5 μM, 1 μM, 5 μM, and 10 μM in aCSF) and dopamine (0 nM, 0.5 nM, 1 nM, 5 nM, 10 nM in aCSF), as previously described (Chefer et al., 2006), at a constant flow rate of 2 μL/min. Microfraction collector sampling (60 μL per sample) was performed every 15 min for 6 h, beginning with a 2 h perfusion with aCSF (pH 7.35, 2 μL/min) before collection. Samples were immediately analyzed for dopamine extracellular levels by high-performance liquid chromatography with electrochemical detection (LC-4C, Basi, IN) (Bustamante et al., 2008) and for glutamate extracellular levels by HPLC (YL9100, Young Lin Instruments, Korea) with fluorometric detection.

Relevant parameters can be extracted from microdialysis curves: the slope represents uptake/clearance efficiency, the x-intercept represents the steady-state extracellular neurotransmitter concentration, and the y-intercept represents basal neurotransmitter efflux/release.

### 2.3 Catalase activity

Catalase enzymatic activity was measured in total liver protein extracts using a modification of the method described by Baudhuin et al. (1964). Briefly, 0.5 µg of total protein was added to a reaction mixture containing 10 mM imidazole buffer (pH 7.2), 6 mM H_2_O_2_, and ultrapure water to a final volume of 200 μL, while the mixture was kept on ice. All samples were analyzed in triplicate. A calibration curve was prepared with H_2_O_2_ concentrations of 7.5 mM, 6 mM, 4.5 mM, 3 mM, 1.5 mM, 0.75 mM, 0.25 mM, and 75 µM. Samples, calibration standards, and the corresponding blanks were incubated for 5 min at 25 °C. The enzymatic reaction was then stopped by adding 115 µL of a 1% Ti(SO_4_)_2_ solution in 2.5 N H_2_SO_4_. The reaction between H_2_O_2_ and titanium forms a yellow complex [Ti(SO_4_)_2_] with a peak absorbance at 410 nm. Catalase specific activity was expressed as the rate of H_2_O_2_ disappearance per minute per µg of total protein.

### 2.4 GLT-1 and DAT exppression

GLT-1 and DAT protein expression were quantified by Western blot in the NAc. Briefly, the antibodies anti-GLT-1 (PA5-19706, ThermoFisher Scientific) and anti-DAT (PA5-110392, ThermoFisher Scientific) were used at a 1:3,000 dilution, whereas anti-β-actin (for loading control, PA1-183, ThermoFisher Scientific) was used at a 1:10,000 dilution in the blots, incubated overnight at 4 °C. After incubation with HRP-conjugated secondary antibody (31,460, ThermoFisher Scientific, 1 h at room temperature, diluted 1:5,000), blotting membranes were revealed with Luminata Western HRP Chemiluminescence Substrate (Merck Millipore). Bands were quantified by densitometry using ImageJ software.

### 2.5 Sample size and statistical analysis

The sample size (n = 6 per group) was based on effect sizes observed in our prior studies using the same UChB model and endpoints (e.g., alcohol intake reduction and changes in GLT-1 expression). These prior data consistently show large effect sizes (η^2^ > 0.4), for which n = 6 provides statistical power >0.8 at α = 0.05. Additionally, this sample size is standard in no-net-flux microdialysis studies due to the method’s high technical complexity.

Data were analyzed using one-way ANOVA followed by Tukey’s *post hoc* test or two-way ANOVA followed by Bonferroni’s *post hoc* test, with the significance level set at p < 0.05.

## 3 Results

### 3.1 Effect of fenofibrate on alcohol consumption during relapse

**FIGURE 1:**
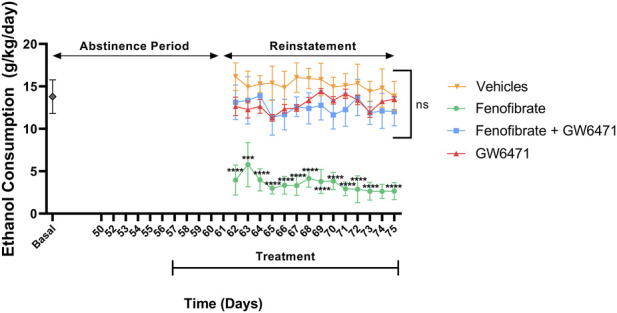
Effect of fenofibrate treatment on alcohol consumption during relapse. Following 45 days of chronic 10% (*v*/*v*) alcohol intake, rats were deprived of alcohol for 14 days, while water was always available. Starting in the last 5 days of deprivation, 4 groups were established, respectively, treated either with a daily dose of: (i) vehicle control (DMSO i.p. + water by gavage); (ii) fenofibrate 50 mg/kg/day + DMSO i.p.; (iii) fenofibrate 50 mg/kg/day plus GW6471 1 mg/kg/day i.p.; or (iv) GW6471 1 mg/kg/day i.p.+ water by gavage. The day after finishing the 14 days of abstinence (which includes the 5 days of drug treatment), the animals were offered the 10% v/v alcohol solution again for an additional 14 days without interruption of the respective treatments, recording consumed volume. Data are presented as means ± SEM; n = 6 rats per experimental group. Two-way ANOVA followed by Bonferroni´s test for multiple comparisons, *** = *p* < 0.001, **** = *p* < 0.0001 vs. Vehicles group; ns = not significant.

Figure 1 shows that after 45 days of voluntary access to a 10% alcohol solution and water, animals consumed an average of 13.8 g of alcohol per kilogram of body weight per day (Basal consumption). Following 14 days of abstinence, which included 5 days of treatment with the respective drugs (Fenofibrate, Fenofibrate + GW6471, GW6471, or Vehicles), on the first day of re-access to alcohol the group treated with fenofibrate alone exhibited a 78% reduction in alcohol consumption compared with the vehicles-treated control group (fenofibrate: 3.5 g/kg/day, vehicles: 16.2 g/kg/day; F (3,20) = 250.4, p < 0.0001). Interestingly, this effect of fenofibrate was blocked when the specific PPARα antagonist was administered simultaneously (Fenofibrate + GW6471), demonstrating that fenofibrate exerts its effects on alcohol intake specifically through activation of its receptor. The group administered GW6471 alone showed consumption levels similar to the Vehicles and Fenofibrate + GW6471 groups, indicating that this antagonist, per se, does not exert any effect on voluntary alcohol intake. Alcohol consumption levels in all groups remained relatively stable throughout the 14 days of re-access and simultaneous treatments, demonstrating that fenofibrate can maintain reduced consumption across the entire post-abstinence relapse period.

### 3.2 Effect of fenofibrate on catalase activity and GLT-1 and DAT expression

**FIGURE 2:**
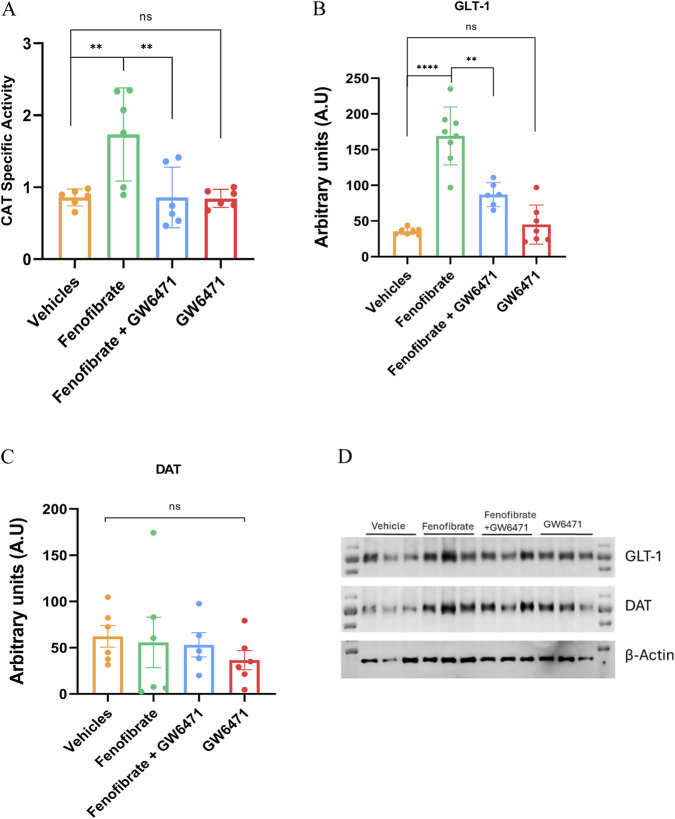
Effect of fenofibrate treatment on hepatic catalase activity, GLT-1, and DAT expression in the NAc. After recording alcohol consumption (Figure 1), catalase activity was measured in liver homogenates from the animals **(A)**. GLT-1 and DAT levels were quantified by Western blot in the animals’ NAc. **(B,C)** show the densitometric quantification of the GLT-1 and DAT bands, respectively, normalized to the β-actin band. **(D)** shows blots from only three animals per group, presented as representative examples on the same blot. Data are presented as means ± SEM; n = 6. One-way ANOVA followed by Tukey´s test for multiple comparisons, ** = *p* < 0.01; **** = *p* < 0.0001; ns = not significant.

To obtain a first approximation of whether peripheral and/or central mechanisms mediate the effect of fenofibrate, we quantified catalase enzymatic activity in the liver (reflecting peripheral effects by increasing blood acetaldehyde levels upon alcohol intake) and GLT-1 and DAT expression in the NAc (which would imply central neurochemical mechanisms). As shown in Figure 2A, fenofibrate doubled hepatic catalase activity compared with the vehicle-treated control group (fenofibrate: 1.73 U/mg protein, vehicle: 0.85 U/mg protein; F (3,20) = 7.4, p = 0.0016). Similar to the effects observed on alcohol consumption (Figure 1), the simultaneous co-administration of GW6471 abolished the effect of fenofibrate on catalase activity, further supporting that the action of fenofibrate is mediated by PPARα activation. Consistent with the results shown in Figure 1, administration of GW6471 alone did affect catalase activity.

We also aimed to investigate whether fenofibrate treatment could exert central effects by analyzing its impact on GLT-1 and DAT protein expression in the NAc. As shown in Figures 2B,D, fenofibrate increased GLT-1 protein expression more than 4-fold (fenofibrate: 169.1 AU, vehicle: 35.6 AU; F (3,20) = 38.67, p < 0.0001). As noted in Figures 1, 2A, co-administration of GW6471 again reduced the effect of fenofibrate, in this case on GLT-1 expression, whereas GW6471 alone once more produced no effect. Regarding DAT protein expression, no significant differences were observed among the four groups (F (3,20) = 0.41, p = 0.75) (Figures 2C,D).

### 3.3 Discrimination of the peripheral and central effects of fenofibrate on alcohol consumption

Since fenofibrate can cross the blood–brain barrier when administered orally (Blednov et al., 2015; Weil et al., 1988), we aimed to evaluate the relative contributions of peripheral and central effects of fenofibrate on alcohol consumption. To this end, PPARα activation in the brain was selectively blocked by intracerebroventricular (i.c.v.) administration of GW6471 while fenofibrate was administered orally.

**FIGURE 3:**
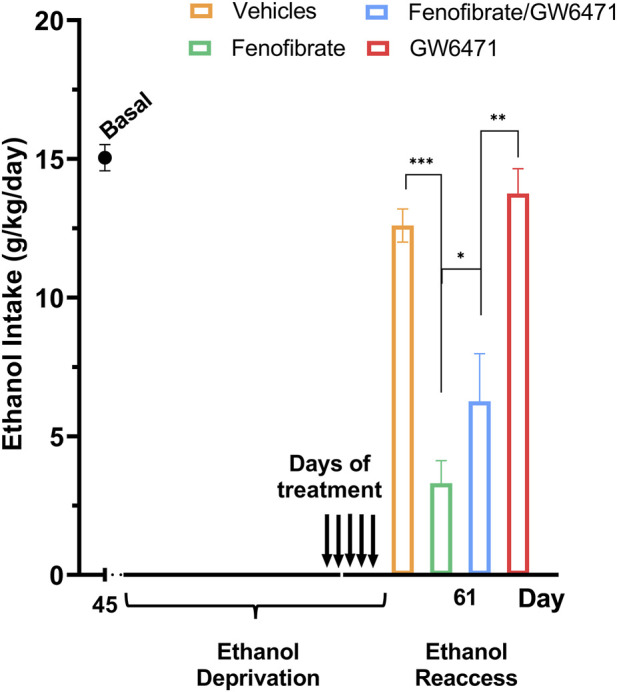
Central blockade of fenofibrate’s effect on alcohol consumption during relapse. Following 45 days of chronic 10% (*v*/*v*) alcohol intake, rats were deprived of alcohol for 14 days. In the last 5 days of deprivation, the 4 groups were treated either with a daily dose of: (i) vehicle control (DMSO i.c.v. + water by gavage); (ii) fenofibrate 50 mg/kg/day + DMSO i.c.v.; (iii) fenofibrate 50 mg/kg/day plus GW6471 1 μg/kg/day i.c.v.; or (iv) GW6471 1 μg/kg/day i.c.v.+ water by gavage. The day after finishing the 14-day period of abstinence (which includes the 5 days of drug treatment), the animals were offered a 10% *v*/*v* alcohol solution again for an additional 24 h, recording the consumed volume. Data are presented as means ± SEM; n = 6 rats per experimental group. One-way ANOVA followed by Tukey´s test for multiple comparisons, * = *p* < 0.05, ** = *p* < 0.01, *** = *p* < 0.001; ns = not significant.

As shown in Figure 3, fenofibrate reduced alcohol intake on the first day of reaccess by 74% compared with vehicle controls (fenofibrate: 3.3 g/kg/day vs. vehicle: 12.6 g/kg/day; F (3,24) = 16.03, p < 0.0001). Unlike Experiment 1, where intraperitoneal GW6471 fully abolished the effect of fenofibrate, i.c.v. co-administration of GW6471 only partially reversed this reduction (fenofibrate + GW6471: 6.3 g/kg/day). Thus, of the 74% decrease produced by fenofibrate, i.c.v. GW6471 reversed 24%, leaving an estimated 50% reduction attributable to fenofibrate’s peripheral (systemic) actions. In other words, approximately one-third of fenofibrate’s effect on alcohol intake is mediated centrally, whereas the remaining two-thirds result from peripheral mechanisms.

### 3.4 Effects of fenofibrate on extracellular glutamate and dopamine levels in NAc

**FIGURE 4:**
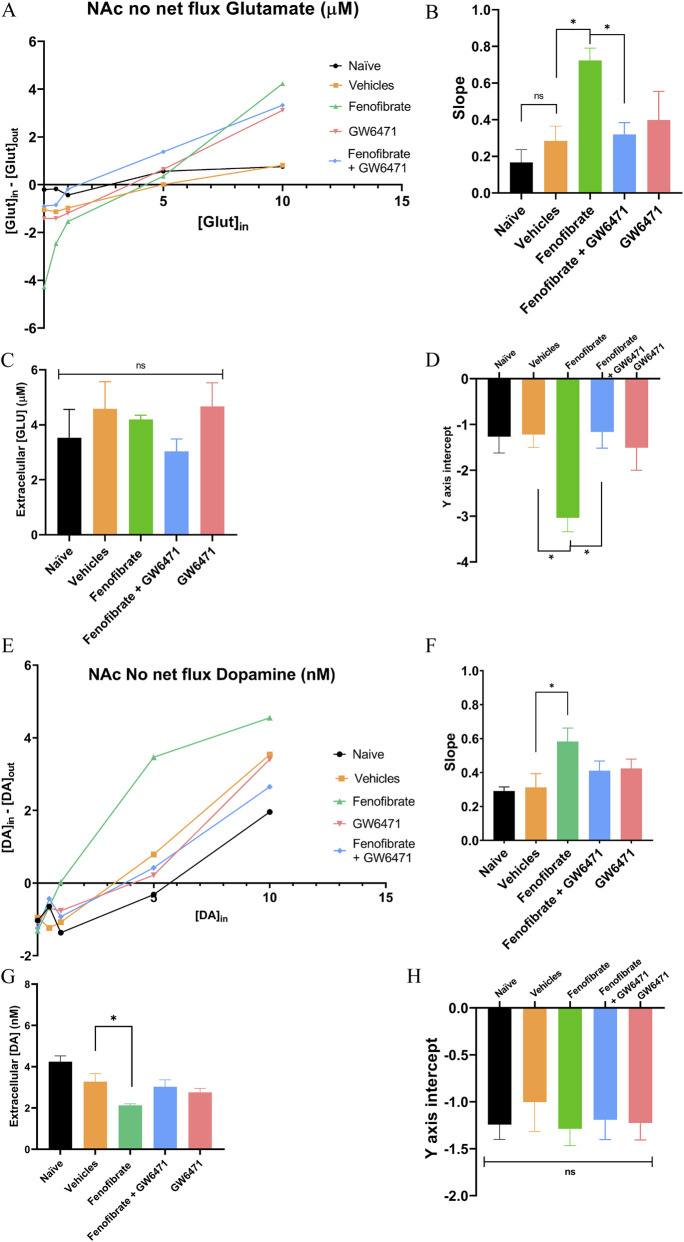
No net flux analysis of glutamate and dopamine in the NAc of UChB rats. Representative curves from no net flux microdialysis of glutamate **(A)** and dopamine **(E)** in the NAc. Average slopes derived from the no net flux microdialysis analysis, representing glutamate **(B)** and dopamine **(F)** uptake rate. Extracellular glutamate **(C)** and dopamine **(G)** levels in the NAc. Y-axis intercept values from microdialysis curves for glutamate **(D)** and dopamine **(H)** n = 6. One-way ANOVA followed by Tukey´s test for multiple comparisons, * = *p* < 0.05; ns = not significant.

To determine extracellular levels of glutamate and dopamine in the NAc as well as their uptake rates, we performed no-net flux microdialysis, in which increasing concentrations of the neurotransmitters were administered to determine the concentration at which there is no net flux to or from the microdialysis probe. At this equilibrium point, the exact concentration of the neurotransmitter in the brain region of interest can be determined (the point where the curves intersect the x-axis). Additionally, the slopes calculated from the microdialysis curves allow estimation of the uptake rate of both neurotransmitters. As shown in Figures 4A,B,E,F, fenofibrate produced a significant increase in the uptake rate of both glutamate and dopamine compared with the control group (glutamate slopes: fenofibrate 0.72 vs. vehicles 0.28; F (4,25) = 4.06, p = 0.02. Dopamine slopes: fenofibrate 0.58 vs. vehicles 0.31; F (4,25) = 4.75, p < 0.01). For glutamate, this observation is consistent with the increase in GLT-1 transporter expression (Figure 2B). However, extracellular glutamate levels were not decreased by fenofibrate treatment (values range between 3–5 μM, F (4,25) = 0.76, p = 0.57) (Figure 4C) (see Discussion). In contrast, extracellular dopamine levels were significantly reduced following fenofibrate administration, which correlates with the observed increase in dopamine uptake (fenofibrate: 2.1 nM vs. vehicles: 3.28 nM; F (4,25) = 6.62, p < 0.01) (Figure 4G).

Similar to what was observed for alcohol consumption (Figures 1, 3), hepatic catalase activity (Figure 2A), and GLT-1 expression (Figure 2B), co-administration of GW6471 blocked the effect of fenofibrate on glutamate and dopamine uptake, as well as on extracellular dopamine levels (Figure 4). For extracellular glutamate levels, this effect did not reach statistical significance, perhaps reflecting that fenofibrate treatment alone did not have any significant effect on this parameter. Another parameter derived from the lines fitted to the microdialysis curves is the y-intercept; this value allows estimation of transporter expression or activity in the membrane (the more negative the y-intercept, the higher the transporter activity). Consistent with Figure 2B, which shows increased GLT-1 expression in the NAc, the microdialysis curves also indicate increased membrane activity of GLT-1 following fenofibrate treatment (Figure 4D). Similarly, and in agreement with Figure 2C, the microdialysis curves do not show that fenofibrate increases the expression and/or membrane activity of DAT (Figure 4H). Although no-net-flux microdialysis showed an increase in dopamine reuptake efficiency after fenofibrate treatment, Western blot analyses of total protein in NAc homogenate revealed no differences in total DAT expression among the groups. These results suggest that fenofibrate does not increase the total amount of DAT but rather modulates its functional localization or activity (e.g., increased DAT membrane expression or post-translational modifications that increase the transport rate).

## 4 Discussion

In previous studies, we showed that fenofibrate reduces voluntary alcohol intake in drinking rats when administered during the chronic consumption phase (Karahanian et al., 2014; Rivera-Meza et al., 2017) or when given only during withdrawal after chronic intake, followed by a 1-h reaccess period (relapse-like alcohol intake) (Ibáñez et al., 2023). In the present work, we demonstrated that initiating fenofibrate treatment during abstinence and continuing throughout the 14-day reaccess phase produced a sustained and marked reduction in relapse-like drinking across the entire reaccess period (Figure 1). This treatment approach enhances translational relevance by avoiding fenofibrate exposure during the chronic—and elevated—alcohol consumption phase, thereby preventing potentially harmful acetaldehydemia beyond toxic thresholds (an effect observed with disulfiram treatment) (Nagaraj et al., 2017), while maintaining treatment before and during the vulnerable relapse window when alcohol intake is strongly reduced.

The magnitude of fenofibrate’s effect observed in the present study (a ∼78% reduction in alcohol consumption during relapse in UChB rats) indicates a robust efficacy within this preclinical model. However, this effect should not be directly equated with the clinical effectiveness of currently approved medications for alcohol use disorder (AUD), as preclinical consumption paradigms and clinical relapse outcomes represent fundamentally different endpoints and are influenced by distinct biological and environmental variables. In clinical settings, medications such as naltrexone, acamprosate, and disulfiram typically produce more modest reductions in relapse risk (Anton et al., 2006; Maisel et al., 2013), reflecting the complexity and heterogeneity of AUD in human populations. Within the context of preclinical studies, however, the magnitude of fenofibrate’s effect is comparable to or greater than that reported for other pharmacological strategies targeting glutamatergic homeostasis, including GLT-1–modulating agents such as ceftriaxone, MS:153, synthetic peptides, or N-acetylcysteine, which typically produce reductions in the range of 50%–60% in alcohol-preferring rat lines (Quintanilla et al., 2020; Rao et al., 2015; Sari et al., 2013; Sari and Sreemantula, 2012). Consistent with our previous findings (Ibáñez et al., 2023), coadministration of the selective PPARα receptor antagonist GW6471 completely blocked fenofibrate’s effect, suggesting that its anti-relapse action is specifically mediated through PPARα activation. Conversely, GW6471 alone had no significant impact on alcohol intake, showing levels comparable to the vehicle group.

Hepatic catalase activity increased ∼2-fold in fenofibrate-treated animals, consistent with our earlier reports (Karahanian et al., 2014; Rivera-Meza et al., 2017). This suggests a peripheral contribution to fenofibrate’s effect via faster ethanol oxidation and enhanced acetaldehyde production, leading to aversive interoceptive cues (i.e., increased acetaldehyde accumulation in blood). However, acetaldehyde-induced aversion alone is unlikely to fully explain the magnitude of the peripheral component. Fenofibrate induces additional metabolic adaptations in the liver, including modulation of alcohol dehydrogenase and peroxisomal β-oxidation (Muñoz et al., 2020), which may influence systemic redox states and peripheral signals that interface with central reward circuits. Thus, the dominant peripheral contribution likely reflects a combination of acetaldehyde-mediated aversion and broader metabolic reprogramming rather than a single mechanism.

Regarding central effects, as in our previous work (Villavicencio-Tejo et al., 2021), fenofibrate increased GLT-1 expression. This effect is also mediated explicitly by PPARα activation, as it was blocked by coadministration of GW6471. The increases in GLT-1 expression and activity (see below) induced by fenofibrate are particularly interesting, since the functionality of GLT-1 can be diminished under conditions with high acetaldehyde levels resulting from chronic alcohol consumption. Studies show that exposure to elevated acetaldehyde impairs GLT-1 transport capacity, disrupting glutamate homeostasis and promoting neurotoxicity (Blanc et al., 1998; Keller et al., 1997). The interplay between increased acetaldehyde and inhibition of glutamate transporters suggests a potential therapeutic mechanism in which GLT-1 upregulation mitigates specific neurotoxic effects of acetaldehyde by reducing excitotoxicity induced by excess glutamate. Fenofibrate did not alter total DAT expression; to our knowledge, there are no reports of PPARα agonists upregulating DAT expression, although they enhance its activity (see below).

Previously, we reported that fenofibrate administration during the withdrawal period after chronic alcohol consumption reduced intake during reaccess by 80% (Ibáñez et al., 2023), and this effect was abolished when GW6471 was administered intraperitoneally simultaneously. It has been reported that GW6471 can reach the brain when administered i.p (Gavzan et al., 2018). In that study, we could not determine whether fenofibrate’s effect occurred centrally and/or peripherally. Therefore, in the present study, we administered GW6471 intracerebroventricularly, thereby blocking fenofibrate’s action only in the CNS, determining the relative contribution of each component to the decrease in relapse drinking. We found that i.c.v. administration of GW6471 partially blocked (33%) fenofibrate’s effect, indicating that fenofibrate acts primarily through peripheral mechanisms (66%). It has been reported that orally administered fenofibrate is unevenly distributed between brain and liver tissue in rodents, with brain concentrations only about 1% of those measured in the liver (Blednov et al., 2015; Weil et al., 1988). Therefore, a much smaller central effect than a peripheral effect would be expected. Nevertheless, it is remarkable that despite this significant tissue-distribution difference, we observed a 33% central contribution. Systemic delivery of PPAR agonists has been shown to influence the CNS, with reports of enhanced cognitive performance (Bhateja et al., 2012), decreased ethanol–induced hyperactivity (Marche et al., 2011), and neuroprotective effects (Bordet et al., 2006). Together, these findings indicate that PPARα agonists exert direct effects in the brain, as proposed for the treatment of neurological diseases (Mandrekar-Colucci et al., 2013; Scheggi et al., 2022).

The no-net-flux microdialysis experiments provide a detailed functional assessment of how fenofibrate modulates neurotransmitter homeostasis in the NAc, revealing a pattern of effects that distinguishes glutamate from dopamine. Fenofibrate increased the slope of the no-net-flux function for both glutamate and dopamine, indicating increased uptake. This steeper slope reflects greater operational efficacy of GLT-1- and DAT-mediated clearance, suggesting that PPARα activation improves the dynamic regulation of extracellular transmitter levels in response to changes in concentration. Despite this shared effect on clearance dynamics, fenofibrate produced divergent effects on the intercept parameters for glutamate and dopamine, indicating distinct mechanisms governing their basal extracellular regulation. For glutamate, fenofibrate increased the y-intercept without altering the x-intercept, consistent with increased net basal efflux or reduced basal uptake, while leaving steady-state extracellular glutamate unchanged. This suggests that fenofibrate modulates the glutamatergic handling at baseline flux—potentially through altered glial transporter activity, trafficking, or presynaptic release probability—without shifting the equilibrium concentration set by tonic glutamate dynamics in the NAc. In contrast, the dopaminergic system showed the opposite effect: while fenofibrate did not alter the y-intercept, it significantly reduced the x-intercept, indicating that the basal efflux was preserved. However, the steady-state extracellular dopamine level was lowered. This finding can be explained by enhanced DAT-mediated clearance in the absence of tonic release modifications. A possible inferred mechanism could be that PPARα activation could promote functional changes in DAT, such as increased membrane trafficking or modulation of transporter kinetics via posttranslational modifications (Foster and Vaughan, 2011; 2017), thereby accelerating dopamine clearance without altering baseline release mechanisms. In any case, this last hypothesis should be tested experimentally. Taken together, the functional and protein expression data suggest a model in which PPARα activation enhances DAT activity. In addition, PPARα activation induces the phosphorylation of β2-nAChRs in the VTA (Melis et al., 2013), leading to decreased dopamine release. Both effects would narrow the dopaminergic dynamic range and could contribute to the reduction in response to motivational stimuli observed in models of alcohol consumption. Additionally, the selective prevention of these effects by GW6471 confirms that they are mediated by PPARα. Collectively, the pattern of changes in slope and intercept indicates that fenofibrate strengthens extracellular neurotransmitter control in the NAc by increasing uptake capacity through increased GLT-1 and DAT activity. However, the consequences for basal extracellular levels depend on the specific transmitter system. Glutamate homeostasis appears buffered at the level of steady-state concentration despite altered basal flux, whereas dopamine homeostasis shifts toward lower tonic levels due to increased clearance efficiency. This transmitter-specific regulation may have important implications for understanding how PPARα activation influences motivational and reward-related circuits implicated in alcohol use disorders. This multimodal action distinguishes fenofibrate from other drugs that act on a single molecular target (e.g., opioid antagonists, GABAergic modulators) and may explain its superior efficacy in animal models. This asymmetry—that is, excitatory homeostasis versus attenuated dopaminergic signaling—provides a molecular mechanism consistent with the reduced response to motivational stimuli observed in models of alcohol consumption and positions the PPARα/GLT-1/DAT axis as a promising therapeutic target for substance use disorders and warrants further investigation into both central and peripheral mechanisms underlying its actions.

Fenofibrate markedly increases GLT-1 expression and uptake slope, yet extracellular glutamate levels remain unchanged. This apparent discrepancy deserves more critical discussion. This interpretation demands careful separation of release and uptake dynamics, because extracellular Glu is shaped by (a) vesicular/synaptic release, (b) non-vesicular leak from extracellular spaces, (c) uptake by transporters (GLT-1 and others), and (d) buffering or metabolism in astrocytes (Milton et al., 2002; Molchanova et al., 2004). No-net-flux measures can shift if concomitant changes in release or buffering offset changes in uptake. This dissociation between uptake capacity and net extracellular levels has been reported in contexts where release changes offset uptake capacity, particularly when no-net-flux slopes are influenced by uptake. Still, basal concentrations are maintained by release dynamics. Supporting evidence from no-net-flux studies shows that chronic intake and withdrawal of alcohol and other drugs of abuse can alter release and uptake independently, yielding similar extracellular Glu levels if opposite directions of change cancel out (Lominac et al., 2012; Pati et al., 2016; Chefer et al., 2009). Additionally, astrocytes contain multiple mechanisms to buffer extracellular Glu beyond transporter uptake, including rapid intracellular metabolism to glutamine and integration into the glutamate–glutamine cycle. If fenofibrate enhances GLT-1 but also bolsters astrocytic enzymes or compartments that transiently buffer or metabolize extracellular Glu, the net extracellular level could remain unchanged even as uptake capacity rises. This perspective is consistent with observations that altering transporter expression does not always shift steady-state extracellular Glu if metabolic sinks are engaged (Demestre et al., 1997; Rappeneau et al., 2016).

We are aware that this study was conducted only with female rats. The converging evidence across murine models and translational data supports a robust view: sex modulates PPARα signaling at multiple levels, including transcriptional networks, post-translational regulation, and tissue- and context-specific responses to dietary (Smati et al., 2022), pharmacological (Cheng et al., 2016), and toxicological challenges (Hirata-Koizumi et al., 2016). Specifically in AUD, sex differences in fibrate effects on alcohol consumption are inconsistently characterized across studies and paradigms. The limited existing literature comparing sex-dependent effects suggests that fibrates have a greater effect in male mice than in females (Blednov et al., 2016a; 2016b), but this effect depends on the type of alcohol exposure (continuous vs. intermittent access). On the other hand, there is converging evidence from both animal models and human studies that sex influences the rates or severity of relapse to alcohol consumption. However, the strength and nature of the effects vary across models, paradigms, and contexts. In animal models, several studies show that females are more prone to relapse than males (Blednov et al., 2016a; Rodd et al., 2005; Sneddon et al., 2022); however, this difference depends on strain, dosing, testing schedule, and whether relapse is measured after repeated deprivation cycles versus a single deprivation event. In the human context, women historically show lower overall AUD prevalence but rising incidence and often different relapse dynamics compared with men; sex differences in relapse vulnerability have been reported in clinical cohorts, with women sometimes exhibiting higher relapse risk in specific contexts and comorbidity profiles, though this is modulated by age, hormonal status, and psychosocial factors (Rodd et al., 2005). These findings have important implications for designing sex-aware dietary interventions, pharmacotherapies (e.g., PPARα agonists), and risk assessments for xenobiotics that activate or repress PPARα signaling.

Finally, from a translational point of view, while rodent studies consistently show that PPARα agonists can reduce alcohol consumption and relapse-like behavior in animals, translating these findings to humans faces several limitations. Fenofibrate has limited brain penetration relative to the liver (Blednov et al., 2015; Weil et al., 1988). The usual effective dose in reducing alcohol consumption in rodents is 50 mg/kg/day (Rivera-Meza et al., 2017). In contrast, in humans, therapeutic doses are equivalent to 145 mg/person/day (Mason et al., 2024). Whether this dose is sufficient to guarantee delivery to the human brain has not yet been studied. Increasing the dose in humans is constrained by the risk of hepatotoxicity, especially in heavy drinkers. This limitation underscores the need for new, effective brain-penetrant PPARα agonists to adequately assess the therapeutic potential of PPARα agonists for AUD. On the other hand, differences in PPAR distribution, signaling, and ligand dynamics between rodents and humans may reduce translational predictability (Matheson and Le Foll, 2020). Also, genetic data suggest that PPAR-related polymorphisms influence alcohol dependence and withdrawal phenotypes, implying heterogeneity may arise in individual response to treatment (Blednov et al., 2015).

Together, these results position the PPARα/GLT-1/DAT axis as a promising yet preliminary target for AUD pharmacotherapy, bridging peripheral metabolic mechanisms with the regulation of central reward circuitry.

## Data Availability

The original contributions presented in the study are included in the article/supplementary material, further inquiries can be directed to the corresponding authors.
